# Supplementary material for: Reporting of mycetoma cases from skin and soft tissue biopsies over a period of ten years: A single center report and literature review from Pakistan
Source: PLoS Negl Trop Dis. 2022 Jul 29;16(7):e0010607. doi: 10.1371/journal.pntd.0010607 (PMC9365187; doi:10.1371/journal.pntd.0010607)
Supplement: Attachment — Submitted filename: Response to reviewers (PNTD-D-22-00093) 14 06 2022.docx [file pntd.0010607.s001.docx]

14^th^ June 2022

To,

**Dr. Claudio Guedes Salgado**

Associate Editor, PLOS Neglected Tropical Diseases

and

**Dr. Ahmed Fahal**

 Deputy Editor, PLOS Neglected Tropical Diseases

**Subject**: Resubmission of manuscript (PNTD-D-22-00093) -(EMID: 71d78df51b96e0d8) titled “*Reporting of Mycetoma cases from skin and soft tissue biopsies over a period of ten 7 years: A single center report and literature review from Pakistan.”*

Dear Dr. Salgado and Dr. Fahal

Thanks for the suggestions to remove separate files of figures and table from the “uploaded re-submission files” on paper titled “*Reporting of Mycetoma cases from skin and soft tissue biopsies over a period of ten years: A single center report and literature review from Pakistan”.*

We have done that as per instruction. I hope we have done it appropriately.

Looking forward for the great news.

With Kind Regards

Afia Zafar

Corresponding author
